# Optimization of hyaluronan-enriched cubosomes for bromfenac delivery enhancing corneal permeation: characterization, ex vivo, and in vivo evaluation

**DOI:** 10.1080/10717544.2022.2162162

**Published:** 2023-01-01

**Authors:** Nabil A. Shoman, Rana M. Gebreel, Mohamed A. El-Nabarawi, Alshaimaa Attia

**Affiliations:** a Department of Pharmaceutics and Pharmaceutical Technology, Faculty of Pharmacy, Ahram Canadian University, Giza, Egypt; b Department of Pharmaceutics, College of Pharmaceutical Sciences and Drug Manufacturing, Misr University for Science and Technology, Giza, Egypt; c Department of Pharmaceutics and Industrial Pharmacy, Faculty of Pharmacy, Cairo University, Cairo, Egypt; d Department of Industrial Pharmacy, College of Pharmaceutical Sciences and Drug Manufacturing, Misr University for Science and Technology, Giza, Egypt

**Keywords:** Hyaluronic, polyvinyl alcohol, cubosomal dispersion, permeation, corneal bioavailability, ocular safety, cataract

## Abstract

To design and evaluate hyaluronan-based cubosomes loaded with bromfenac sodium (BS) for ocular application to enhance the corneal permeation and retention in pterygium and cataract treatment. BS-loaded cubosomes were prepared by the emulsification method, employing 2^3^ full factorial design using Design-Expert® software. Glycerol monoolein (GMO) and poloxamer 407 (P407) as lipid phase and polyvinyl alcohol (PVA) as stabilizer were the used ingredients. The optimized formulation (OBC; containing GMO (7% w/w), P407 (0.7% w/w) and PVA (2.5% w/w)) was further evaluated. OBC had an entrapment efficiency of 61.66 ± 1.01%, a zeta potential of −30.80 ± 0.61 mV, a mean particle size of 149.30 ± 15.24 nm and a polydispersity index of 0.21 ± 0.02. Transmission electron microscopy confirmed its cubic shape and excellent dispersibility. OBC exhibited high stability and no ocular irritation that was ensured by histopathology. Ex vivo permeation study showed a significant increase in drug deposition and permeability parameters through goat cornea, besides, confocal laser microscopy established the superior permeation capability of OBC, as compared to drug solution. In vivo pharmacokinetics in aqueous humor indicated higher AUC_0-tlast_ (18.88 µg.h/mL) and mean residence time (3.16 h) of OBC when compared to the marketed eye drops (7.93 µg.h/mL and 1.97 h, respectively). Accordingly, hyaluronan-enriched cubosomes can be regarded as a promising carrier for safe and effective topical ocular delivery.

## 1. Introduction

The effective ocular drug delivery has stayed an unmet challenge attributable to the inimitable anatomical and physiological limitations of the eye such as reflex blinking, low drug permeation and tear discharge, making it difficult to achieve an effective drug concentration within the targeted ocular tissues. Furthermore, drugs administered topically suffer from a low ocular bioavailability reaching < 5% (Akhter et al., 2022). Lipid-based nanosystems were among various novel delivery systems that have been investigated to enhance ocular drug bioavailability (Barkat et al., 2020). Lipid-based nanosystems have been recently evolved as effective platforms attaining a superior ophthalmic therapeutic outcome. Cubosomes, as a novel targeted drug delivery system, have been widely used in the management of several diseases with different routes involving oral, ocular, intravenous and percutaneous routes of administration (Garg et al., 2021).

Cubosomes are thermodynamically stable liquid crystalline nanosystems comprising a highly convoluted, continuous lipid bilayer enclosing two nonintersecting water channels (Muller et al., 2010). They can be formulated by the spontaneous self-assembly of amphiphilic/polar lipids such as glycerol monooleate (GMO) or phytantriol with suitable a stabilizer in presence of water (Rizwan et al., 2013). GMO cannot form a stable emulsion without the presence of a stabilizer such as Poloxamer 407 (P407), a nonionic tri-block copolymer, containing both hydrophilic (polyethylene oxide (PEO)) part and hydrophobic (polypropylene oxide) part. P407 is the most widely used surfactant in the preparation of cubosomes as it would sterically stabilize the cubic phase and preserve the inner colloidal stability of dispersed liquid crystalline particles (Huang et al., 2017). Cubosomes have many advantages that make them appealing as a promising novel ocular drug delivery system. Biodegradable lipids that are included can form nanoparticles with higher corneal penetrability, drug protection from physical and chemical degradation, controlled drug release, biocompatibility, and biodegradability. Moreover, they could exhibit higher physical stability than liposomes owing to the strong electrical repulsive forces and the large ratio of lipid bilayer (Gaballa, El Garhy, Abdelkader, et al., 2020). They have larger specific surface area, bio-adhesion, good flowability, low viscosity, ease of preparation with simple techniques and economics. Their structure enables the encapsulation of various hydrophilic, hydrophobic, or amphiphilic drug molecules (Rarokar et al., 2016).

Non-steroidal anti-inflammatory drugs (NSAIDs) are used clinically to reduce the inflammatory process owing to the manipulation of ocular structures like, surgery, trauma, infections, among others. As a cyclooxygenase (COX-1 & COX-2) inhibitor, NSAIDs have been widely considered as an effective alternative to ocular corticosteroids for the relief of ocular inflammation and post-operative pain. Bromfenac sodium (BS) is the first and only topical ophthalmic NSAID with a once-daily dosing regimen approved by the US Food and Drug Administration for the management of pain and inflammation associated with cataract surgery (Cable 2012). It has good ocular penetration with insignificant systemic reactions following topical administration. Bromfenac, like other NSAIDs, is a weakly acidic drug. Reducing the pH formulation increases the unionized fraction of the drug, which, in turn, improves its ocular penetration (Hoffman et al., 2016). It is a very potent (COX-2) inhibitor of prostaglandin production that improves the inflammation signs induced by eye dryness in patients with of Dry eye syndrome (DES) (Fujishima et al., 2015). DES is a multifactorial disorder of the preocular tear film and ocular surface characterized by pain, visual disturbance, and tear film instability. DES affects between 5% and 34% of people, with additional symptoms including redness, burning, itching, sensation of foreign objects, pruritus, stinging and light sensitivity (Huynh and Priefer 2020). In recent decades, the most commonly used therapy is the use of artificial tears made up of sodium hyaluronate, polyvinyl alcohol, povidone, and cellulose derivatives (Fezza 2018).

Hyaluronan (HA) is a member of the family of glycosaminoglycans and owes its efficacy to hyaluronic acid that consists of repeating disaccharide units of N-acetyl-D-glucosamine and sodium-D-glucoronate. It is a natural carbohydrate biopolymer in physiological tear fluid that has great moisturizing and mucus-layer adhesion abilities. Therefore, the adverse effects related to its administration should be minimal (Ángeles and Nešporová 2021). HA has emerged as an option in artificial tear therapy and plays an important role in organizing and maintaining tissues (Ang et al., 2017). It has also been reported to display anti-inflammatory, immunosuppressive and wound healing-promoting effects (Zamboni et al., 2018). HA has been established to be useful in the treatment of DES due to its ability to retain water and allowing lubricant properties to the ocular surface. Accordingly, there is an improvement in the tear film thickness and the ocular surface index as well as a reduction in the friction during blinking and ocular movements (Cagini et al., 2021). The treatment with bromfenac 0.09% and HA 0.4% ophthalmic solutions in a combination therapy for 3 weeks reduced the presentation of clinical signs associated with superficial ocular inflammation in patients with pterygium I–III (Chávez-Mondragón et al., 2019).

Apart from formulation and optimization of pharmaceutical drug delivery systems with few experiments, computer design software was employed as an economical approach. This is tailored by selecting the limits of independent variables to be included and setting certain constraints for the measured parameters, based on the aims of the study (Lewis et al., 1998).

This paper studies the rational combination of HA and PVA as dual functioning mediator for enhancing the physical stability of BS-loaded cubosomes and investigating their potential use for improving ocular delivery and the bioavailability of the drug. Therefore, the objective of our study is to design a novel vehicle based on cubosomes and enriched with hyaluronan as an ophthalmic delivery system using PVA to augment the efficacy, stability, and bioavailability. To explore the efficacy of the optimized formulation, the ex-vivo corneal penetration studies for cubosomal dispersion were performed as well as in vivo assessment of ocular bioavailability was compared with that of commercial eye drops.

## 2. Materials and methods

### 2.1. Materials

Bromfenac sodium (BS) was kindly supplied by Eva Pharma, Egypt. Glycerol monoolein (GMO), Poloxamer 407 (P407) and polyvinyl alcohol (PVA, molecular weight 13,000–21,000) were purchased from Sigma Chemical Company, USA. Hyaluronan (HA, molecular weight ∼10,000 daltons) was purchased from Singclean Medical Products Co., Ltd, Hangzhou, Zhejiang, China. Absolute alcohol, Methyl alcohol, Disodium hydrogen phosphate, Potassium dihydrogen phosphate and Sodium chloride were purchased from El-Nasr Pharmaceutical Chemicals Company, Cairo, Egypt. Methanol HPLC grade, Orthophosphoric acid and Ammonium dihydrogen orthophosphate were obtained from Elnasr abo-zabal, Egypt. Milli-Q water was used for HPLC mobile phase.

### 2.2. Experimental design construction

A 2^3^ full-factorial design was applied for statistically optimizing the factors for the preparation of BS-loaded cubosomes using Design-Expert® version 7.0.0 software (Stat-Ease, Inc., Minneapolis, Minnesota, USA). In this design, the three variable factors: GMO concentration (A), P407 concentration (B), and PVA concentration (C) were assessed. Each factor was set at two levels, which corresponds to the values in Table 1. The BS entrapment efficiency % (EE%) (Y_1_), zeta potential (Y_2_), particle size (Y_3_), and polydispersity index (Y_4_) were considered as response parameters. Eight different systems representing all possible combinations between factors were proposed by design software (Table 2). Formulations were performed twice in two separate replicates to minimize the error in the factorial design.

**Table 1.** Independent and dependent variables with their respective constraints of the 2^3^ full factorial design for the preparation of BS-loaded cubosomal dispersion formulae.

Factors (Independent Variables)	Levels	
	Low	High
A: GMO concentration (% w/w)	3.5	7
B: P407 concentration (% w/w)	0.35	0.7
C: PVA* concentration (% w/w)	2.5	5
**Responses (Dependent Variables)**	**Desirability Constraints**	
Y_1_: EE (%)	Maximize	
Y_2_: ZP (-mV):	Maximize (as absolute value).	
Y_3_: PS (nm)	Minimize	
Y_4_: PDI	Minimize	

* Concentration with respect to dispersed phase.

**Abbreviations:** BS, bromfenac sodium; GMO, glyceryl monoolein; P407, poloxamer 407; PVA, polyvinyl alcohol; EE, entrapment efficiency; ZP, zeta potential; PS, mean particle size; PDI, polydispersity index.

**Table 2.** (I) Experimental runs, independent variables and their responses using 2^3^ full factorial design for BS-loaded cubosomal dispersions, and (II) Output statistical data of the design.

	Factors			Responses			
	A: GMO conc	B: P407 conc	C: PVA conc	Y_1_: EE	Y_2_: ZP	Y_3_: PS	
	% w/w	(% w/w)	(% w/w)	(%)	(-mV)	(nm)	Y_4_: PDI
C1	7	0.7	2.5	61.66 ± 1.01	30.80 ± 0.61	149.3 ± 15.24	0.21 ± 0.02
C2	7	0.7	5	65.45 ± 0.65	27.40 ± 0.35	179.77 ± 21.33	0.37 ± 0.01
C3	7	0.35	2.5	70.46 ± 0.89	25.17 ± 0.55	179.67 ± 1.51	0.27 ± 0.01
C4	3.5	0.7	2.5	40.52 ± 1.16	27.53 ± 0.23	121.33 ± 19.98	0.43 ± 0.01
C5	3.5	0.35	5	51.61 ± 0.65	26.07 ± 0.46	211.03 ± 7.51	0.28 ± 0.01
C6	7	0.35	5	65.70 ± 0.78	27.70 ± 0.46	132.47 ± 3.48	0.48 ± 0.03
C7	3.5	0.35	2.5	53.29 ± 0.97	25.93 ± 0.38	167.97 ± 3.66	0.27 ± 0.01
C8	3.5	0.7	5	54.68 ± 1.25	23.33 ± 0.57	260.37 ± 15.35	0.44 ± 0.02
C1 without HA	7	0.7	2.5	47.96 ± 0.37	5.48 ± 0.88	170.8 ± 4.81	0.19 ± 0.02
**II**							
Adjusted R^2^				0.9894	0.9426	0.9115	0.9639
Predicted R^2^				0.9844	0.9154	0.8696	0.9468
Adequate precision				56.608	27.073	18.936	27.010
Significant factors				A, B, C	A, B, C	A, C	A, B, C

**Abbreviations:** BS, bromfenac sodium; HA, hyaluronan, GMO, glyceryl monoolein; P407, poloxamer 407; PVA, polyvinyl alcohol; EE, entrapment efficiency; ZP, zeta potential; PS, mean particle size; PDI, polydispersity index.

### 2.3. Preparation of BS-loaded cubosomal dispersions by emulsification method

Glycerol monoolein (GMO), and poloxamer 407 (P407), as the oil phase, were melted in a beaker on a hot plate and magnetic stirrer (MS-300HS, Misung Scientific Co., Korea) adjusted at 70 °C. Bromfenac Sodium (0.09% w/v), hyaluronan (0.4% w/v), and polyvinyl alcohol (PVA) were dissolved in deionized water and heated to 70 °C. After that, the aqueous phase was added to the melted lipid mixture which is mechanically stirred at 500 rpm for 2 h to obtain fully homogeneous cubosomal dispersions. Afterwards, the nano-dispersions were subjected to homogenization (Homogenizer Silent Heidolph Crusher, Schwabach, Germany) at 15,000 rpm for 1 min. Finally, the dispersions were kept refrigerated at (2–8 °C) in amber glass vials for more investigations (Salah et al., 2017). The composition of different suggested BS-loaded cubosomes (C1 to C8) and their measured responses were presented in Table 2.

### 2.4. Characterization of the prepared BS-loaded cubosomal dispersions

#### 2.4.1. Determination of entrapment efficiency percentage (EE%)

The vesicular dispersion for the prepared formulae (about 1 mL) was centrifuged at 20,000 rpm for 1 h at 4 °C (cooling ultracentrifuge, Sigma 3 K 30, Germany). The supernatant was separated, followed by suitable dilution, to evaluate the unentrapped amount of drug in cubosomes by measuring the absorbance spectrophotometrically at predetermined λ_max_ 268.2 nm (Shimadzu UV1650 Spectrophotometer, Kyoto, Japan). EE% was then mathematically calculated using a previously established calibration curve by applying the following equation:

(1) EE% = (Total amount drug– Unentrapped drug) x 100/Total amount drug

Triplicates for each cubosomal dispersion were performed.

#### 2.4.2. Determination of particle size (PS), polydispersity index (PDI) and zeta potential (ZP)

Dynamic light scattering technique was used to measure the PS (Z-average) and PDI at 25 °C using Zetasizer Nano ZS (Malvern Instruments, UK). The prepared dispersions were appropriately diluted before every measurement for obtaining the optimal intensity of light scattering. The ZP evaluation was carried out using the same instrument by tracking the mobile particles in the electric field. The measurements were performed after dilution (1:100). The recorded results were the averages of triplicate experiments ± standard deviation (SD).

### 2.5. Optimization of BS-loaded cubosomal dispersions

System optimization was performed to achieve formulation with the highest EE% and ZP values as well as the least PS and PDI (Table 1). The selection relied on the desirability function which permitted the investigation of all the constraints and responses together, simultaneously, then the formula with the highest desirability value (near to one) was chosen. Finally, the suggested optimized formula was prepared and evaluated in triplicate then compared with the predicted responses to check the accuracy of the model performance. To confirm the accuracy of the model performance, the observed responses had been compared with the predicted responses and should lie within the 95% prediction interval. For comparative purposes, to demonstrate the influence of hyaluronan on the stability of the BS-loaded cubosomal dispersions, the optimal formula was prepared by similar means; but lacking the hyaluronan. The EE%, ZP, PS and PDI of such formula were evaluated in triplicates with comparing the mean values using Student t test (at α = 0.05).

### 2.6. Further in vitro characterization of optimal bromfenac cubosomes (OBC)

#### 2.6.1. Short term physical stability study

The OBC dispersion was stored in sealed glass vials (30 ml) at 4 °C and 25 °C for 90 days to investigate the physical stability of the formulation. Samples from each dispersion were taken at 0, 45 and 90 days. At the end of the storage period, EE%, PS, ZP, and PDI were determined as mentioned earlier and compared with the freshly prepared one, using Student t test (at *p* ≤ 0.05). In addition, the system was visually inspected for aggregation, drug leakage or any other physical change (Albash et al., 2019).

#### 2.6.2. Refractive index and pH measurement

The light refractive index of the OBC formula was measured at 25 °C using Hilger and Watts refractometer (model-46.17/63707, Hilger and Watts Ltd., London, UK), to ensure there is minimal or no blurring in vision caused by the formulation. While the pH of the OBC formula was determined using a calibrated pH-meter (Sartorius, Germany) at room temperature to ensure its ocular tolerance. The measurements were done in triplicate and the results were their mean ± SD.

#### 2.6.3. Morphology of OBC

The morphology was examined for the optimized formula using a transmission electron microscope (TEM) (JEM-1230, Joel, Tokyo, Japan). The formula was diluted then placed on the top of a carbon-coated grid and negatively stained with a 2% w/v phosphotungstic acid solution. The sample was left to dry at room temperature before being observed in the microscope.

### 2.7. Ex vivo and in vivo characterization of OBC

All ex vivo and in vivo experimental procedures were performed in accordance with the ethical guidelines of Research Ethics Committee, Faculty of Pharmacy, Cairo University (REC-FOPCU) (PI 3024).

#### 2.7.1. Transcorneal drug permeation studies

Ex vivo transcorneal permeation profile of OBC dispersion compared to the marketed Bromoflam^®^ eye drops (0.09% w/v) were assessed on excised goat cornea (*n* = 3) using a modified Franz diffusion cell with a diffusion area of 0.785 cm^2^. The fresh whole eyeballs of goats were obtained from a local slaughterhouse and were inspected for any damage, then soaked in normal saline, and transported to the laboratory in a cold chain. Corneas with the surrounding scleral tissue (5–6 mm) were carefully preserved in freshly prepared simulated tear fluid (STF; pH 7.4) (Tayel Saadia Ahmed et al., 2013). The fresh goat corneal tissues were fixed between the donor and receptor chambers. An amount of 1 mL of OBC dispersion and the marketed Bromoflam® eye drops, equivalent to 0.9 mg of bromfenac, were accurately measured and placed in the donor cells. The receptor compartment was filled with 50 mL of STF solution (pH 7.4) containing 25% methanol to ensure sink condition and maintained under magnetic stirring at 50 rpm. Every hour, 0.5 mL of permeation media was withdrawn, and an equal volume of fresh media was added to the receiver cell. The samples were filtered through a 0.45 µm membrane and analyzed using a validated HPLC method, for which the mobile phase was composed of methanol: ammonium dihydrogen orthophosphate buffer (pH 6) (60:40, v/v). The drug was separated into column C18 phenomenex luna using a flow rate of 1 mL/min. The retention time of the drug was nearly 3.64 min (Kiran and Raja 2018). The experiments were performed in triplicate. The amount of drug permeated through the corneal epithelium was plotted versus time. The apparent corneal permeability coefficient (cm/h) for both formulations was calculated according to the equation (Abdellatif et al., 2022):

(2) $$Kp=\;\frac{J_{ss}}{C_{0\;}}$$

where J_ss_ (steady stat flux) was the slope of the linear portion (µg/h cm^2^) and C_0_ was the initial drug concentration (µg/cm^2^). Moreover, the enhancement ratio was determined by dividing the J_ss_ of the OBC dispersion by the J_ss_ of the control Bromoflam® drops. All measurements were done in triplicate (mean ± SD). The results were analyzed statistically by applying a one-way ANOVA test using IBM SPSS Statistics 20 (Armonk, NY, USA) and the significant differences were considered when P-value < 0.05.

Finally, the pretreated corneal tissue was carefully washed three times with STF to clear loosely attached formulation and free bromfenac drug then soaked in methanol (20 ml) overnight. The recovered tissues were sonicated for 15 min in a bath sonicator (Elmasonic S 60 H, Elma, Bangkok, Thailand) to recover the drug retained in the corneal tissue in each sample. Finally, aliquots were withdrawn, filtered through a 0.45 µm membrane, and analyzed using a validated HPLC method as mentioned before to measure the amount retained in the tissue after the permeation investigation.

#### 2.7.2. Confocal laser scanning microscopy (CLSM)

To determine the permeation of the optimized formulation through different corneal layers, the formula was prepared as previously reported, except that the drug was replaced 0.1% w/w Rhodamine B to be imagined under CLSM (Sayed et al., 2021). The goat cornea was mounted in diffusion chambers with the previous aspects of the ex vivo permeation study. To mimic the administration of optimized formula in contact with the eye surface, rhodamine-loaded nanovesicles was applied on the corneal surface and remained for 6 h. Longitudinal sections retained in paraffin wax and cut into sections using a microtome (Rotary Leica RM2245; Leica Biosystems, Wetzlar, Germany) was observed for fluorescence in the corneal tissues. The slides were visualized employing an inverted microscope (LSM 710; Carl Zeiss, Oberkochen, Germany). The cornea was optically scanned under a 40× objective lens (EC-Plan Neofluar 63x/01.40 Oil DICM27). Confocal images were supplied by the LSM Image Browser software, release 4.2 (Carl Zeiss Microimaging GmbH, Jena, Germany). Several sections were selected in corneal tissues and, the intensity of light is analyzed. The experiment was carried out in three trials ± SD.

#### 2.7.3. In vivo assessment of ocular bioavailability

For pharmacokinetic analysis, male healthy albino New Zealand rabbits (2–3 kg) were randomly divided into the following two groups, each group were containing five animals (Xing et al., 2021). The first group was treated with Bromoflam^®^ eye drops and the other group was administered the OBC formulation. The rabbits were anesthetized with sodium pentobarbital (30 mg/kg) injected into the marginal ear vein and secured with a rabbit bag throughout the experiment. Following the administration of 50 μL commercial or OBC formulation (both 0.09 mg/mL), 20 μL aqueous humor samples were collected by 1 mL insulin needle after 0.5, 1, 2,3, 4, 8, and 10 hours and placed in a centrifuge tube. Protein was precipitated by vortex mixing with 100 μL 6% perchloric acid-methanol solution.

Precipitated protein was removed by centrifugation at 4000 rpm for 10 min, and the concentration of drug in the supernatant was determined by HPLC 1200 series with quaternary pump, a manually rheodyne injector with 20 µL sample loop, and a phenomenex luna-column (C18, 250 mm × 4.6 mm I.D. 5 µm size). The mobile phase was methanol and buffer (0.5% Ammonium dihydrogen orthophosphate) in a ratio of 60:40 v/v. Buffer was prepared by dissolving 5 gm of ammonium dihydrogen orthophosphate in 1 L deionized water with pH 5.5–6 with orthophosphoric acid. The injection volume was 20 µL and the flow rate was 1.0 mL/min. The column temperature was maintained at 25 °C. The eluent was detected at 288 nm (Kiran and Raja 2018).

The pharmacokinetic parameters were calculated by a non-compartmental pharmacokinetic model using the PK solver program. Statistical analysis was performed using IBM SPSS Statistics 20 (Armonk, NY, USA) and the differences were considered to be significant at P-value <0.05.

#### 2.7.4. Histopathological examination for ocular irritation

A histopathological study was performed to evaluate the safety of OBC formulation to the ocular tissues. The study was done using male healthy albino New Zealand rabbits (*n* = 5, 2–3 kg). Each rabbit was kept in a separate cage under standard conditions of humidity, temperature and light/dark cycling. Standard dry food and water were given ad libitum. A slit lamp was utilized to investigate frequently the rabbits’ eyes for any blemishes or irritation throughout the study.

About 50 µL of OBC dispersion was topically applied on the one eye of each rabbit, whereas the same volume of sterile normal saline (negative control) was applied on the other eye. The treatments were applied two times a day for 10 days (Abdelbary et al., 2016). The rabbits will be anesthetized using injection of thiopental (20 mg/kg, IV). After scarification by decapitation, the corneas were excised from the separated eyes and carefully rinsed using normal saline to prevent their damage followed by storage in 10% v/v neutral buffered formalin till processed (Nair et al., 2021). Solid paraffin blocks were prepared, and thin sections were obtained using a microtome. The specimens were stained with Harris’s hematoxylin and eosin, and examined using a digital optical microscope (Leica, Cambridge, UK) (Mohamed et al., 2022).

## 3. Results and discussion

### 3.1. Statistical evaluation of the experimental design

Design-Expert software was used to evaluate the effect of 3 independent variables, each at 2 different levels, on BS-loaded cubosomal dispersions according to 3^2^ full factorial design. The independent variables under investigation were GMO concentration (A), P407 concentration (B), and PVA concentration (C), while BS entrapment efficiency % (EE%) (Y1), zeta potential (Y2), particle size (Y3), and polydispersity index (Y4) were selected as the dependent variables reflecting the nanoparticles’ characteristics (Table 1). The analysis of variance (ANOVA) test was carried out for each response and the p-value <0.05 was considered statistically significant.

**Figure 1.**
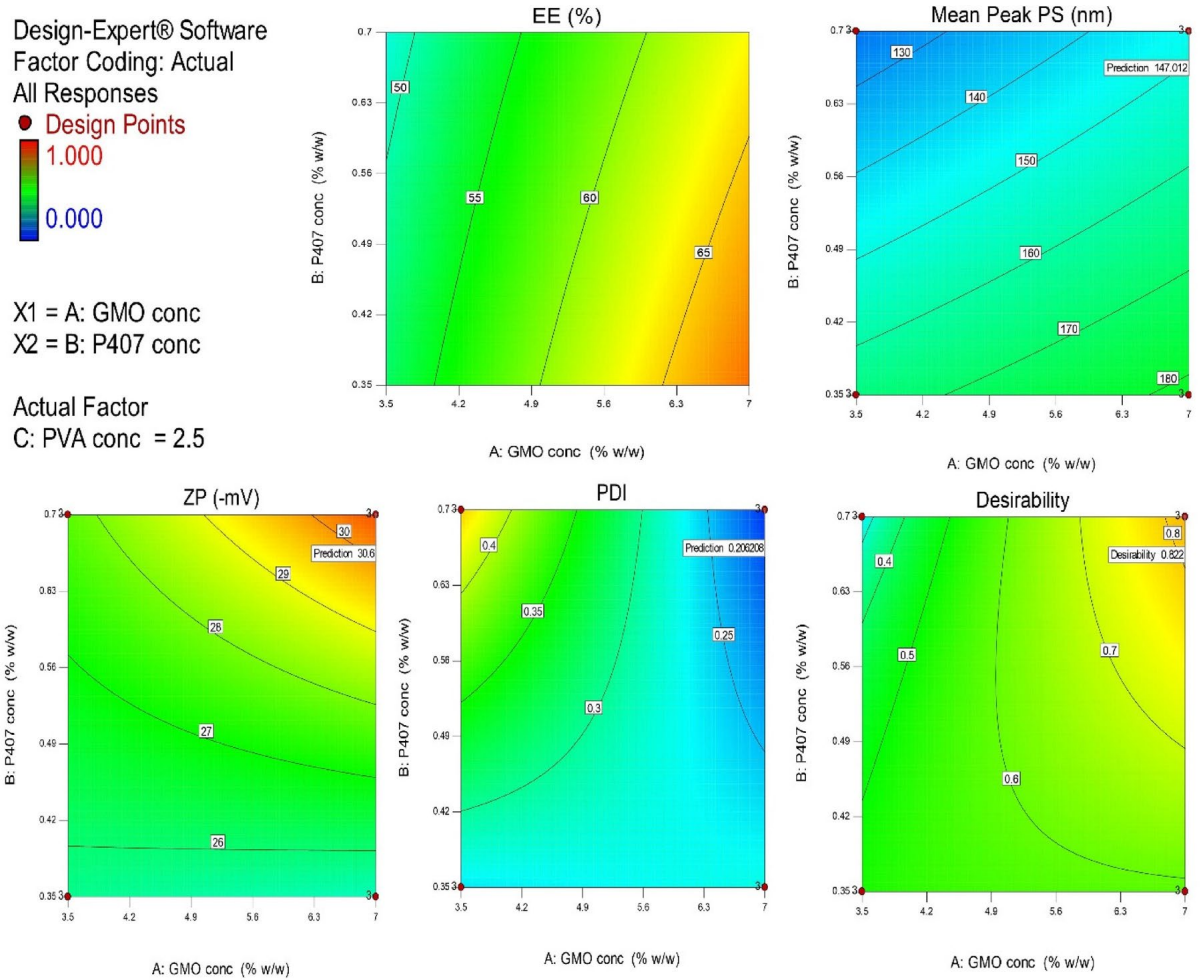
Contour diagram for the joint effect of GMO concentartion (A), and P407 concentartion (B), with an actual factor PVA concentaration (C) to simultaneous prediction of all responses; entrapment efficiency percentage (EE %) (a), mean particle size (b), zeta potential (c), polydispersity index (d), and desirability (e) of bromfenac-loaded cubosomal dispersions.

Adequate precision value confirmed the ability to navigate the design space when the measured signal to noise ratio is greater than 4, which was recognized in all responses (Table 2). The lack of fit was non-significant. Also, the predicted R^2^ and the adjusted R^2^ values were in a reasonable agreement for all responses. The surface plots represented the effect of significant independent variables on all responses and the possible predicted values from the model (Figure 1).

### 3.2. Effect of formulation variables on the EE%

The EE% for the prepared BS-loaded cubosomes ranged between 40.52 ± 1.16 and 70.46 ± 0.89% as shown in Table 2. The relatively low encapsulation efficiency of cubosomes for bromfenac, a hydrophilic drug, was attributed to the limited encapsulation capacity of the water channel in the cubosomal nanoparticles (Nasr et al., 2015). These conditions might favor the rapid leakage of the drug from the aqueous channels to the surrounding aqueous phase during the preparation and centrifugation processes (Bei et al., 2010). Statistical analysis using ANOVA revealed that all model terms (A, B, C, AB, AC, BC) had a significant effect (*P* < 0.05) on EE%. The polynomial equation in terms of coded factors for EE% was:

(3) $${\left(EE\right)}^{2.78}=+84364.41\;+29806.15\;A\;-8409.05\;B\;+3213.46\;C\;-2518.58\;AB\;-5019.94\;AC\;+10629.41\;BC$$

Consequently, from the regression coefficients, input factors such as concentrations of GMO (A) and PVA (C) exhibited a positive effect and improved encapsulation efficiency. Higher amounts of GMO led to an increase in EE%, which may be explained in terms of creating a lipid coat on the exterior of the polymeric core, where the GMO layer provided a denser shell around the polymer core that hindered the leakage of the drug (Khan et al., 2019; Abd-Elsalam and Ibrahim 2021).

Considering PVA, it could be explained by the increase in viscosity of the dispersion viscosity with increasing the PVA concentration that accompanied with an escalation in the rigidity of nanoparticles reducing the leakage of encapsulated drug from dispersions during the ultracentrifuge (Sahoo et al., 2002; Nawaz et al., 2021). On the contrary, P407 concentration (B) had negative effect and increasing this input parameter led to decline in drug entrapment efficiency. When the content of P407 increased, the ability of the cubic structure to hold the drug decreased possibly due to the increased hydrophilicity of the cubic structure and escape of the drug into the outer aqueous environment together with P407 (Lai et al., 2010). These results are in accordance with previous findings (Hosny 2020).

### 3.3. Effect of formulation variables on ZP (-mV)

Zeta potential measurement of the prepared formulations was established to determine the surface charge of the prepared nanoparticles to predict the long term stability of the BS-loaded cubosomal dispersions (Pal et al., 2011). Generally, the cubosomal systems are less colloidally stable than ordinary emulsion in aqueous media, thus the addition of a steric stabilizer (P407) and polymers such as PVA and hyaluronan were required to retain stable dispersions (Chong et al., 2015). It is reported that incorporating hyaluronan into nanoparticles led to highly negative ZP. This could be explained in the light of the fact that the incorporation of negatively charged hyaluronan causes extensive adsorption on the surface of these nanoparticles (Wadhwa et al., 2010; Fahmy et al., 2021). It is documented that the ZP value at least equals to ± 30 mV of the prepared cubosomes was essential to ensure the stability of colloidal dispersions (Vasanth et al., 2020). This value provides an electrostatically thick diffusion layer that acts as a boundary preventing dispersed particles from being aggregated (Sherif et al., 2014). All ZP measurements revealed that all formulated BS-loaded cubosomes had a negative charge. The ZP values ranged between − 23.33 ± 0.57 to − 30.80 ± 0.61 mV which is in good matching with the theoretical accepted values of zeta potential (Table 2) (Thomas and Viswanad 2012).

Statistical analysis using ANOVA revealed that all model terms (A, B, C, AB, AC, BC) had a significant effect (*P* < 0.05) on ZP. The final equation illustrating the impact of the relative proportion of the studied factors on EE% in terms of coded factors was:

(4) ZP= +26.74 +1.025 A +0.525 B −0.617 C +0.808 AB +0.4 AC −1.28 BC

Accordingly, from the regression coefficients, input factors such as concentrations of GMO (A) and P407 (B) exhibited a positive effect and so that high negative charge of ZP point to high repulsion within system, which by turn means higher stability (Fouda et al., 2018).

Regarding factor A, the increase in ZP value might be attributed to the presence of two hydroxyl groups of the glycerol moiety that confer to the negative polar head (Hundekar et al., 2014; Mohyeldin et al., 2016) besides, the ionized negatively charged carboxylic groups in the free fatty acids of GMO, confirming the adsorption of GMO on the exterior of the polymeric core due to the lipophilic nature of these acids (Han et al., 2010; Nazaruk et al., 2017). Considering P407 concentration (factor B), the increase in negative charge owing to the functional groups, the ethylene oxide units and the hydroxyl groups, of P407 that are anchored on the surface of the cubosomes (Tayel et al., 2016; Salah et al., 2017). Also, P407 has a stabilizing effect that creates not only an effective electric repulsion inhibiting the aggregation of nanoparticles (Elnaggar et al., 2015), but also provides an electrostatic barrier between cubosomal nanoparticles, preventing near particles from colliding and maintaining the dispersed particles in a stable form (Elnaggar et al., 2015; Gaballa, El Garhy, Abdelkader 2020).

On the other hand, PVA concentration (C) had a negative effect so that decrease in this input parameter which, in turn, hinders aggregation of particles. The role of PVA is to coat the surface charge of nanoparticles. Thus, the higher ZP values of nanoparticles prepared with 2.5% PVA could be attributed to the lower amount of residual PVA with less shielding effect expected in addition to the more carboxyl groups accessible for ionization as compared to the nanoparticles prepared with 5% PVA (Sahoo et al., 2002).

### 3.4. Effect of formulation variables on PS (nm)

The particle size of the nanodispersion is a significant characteristic which may affect drug penetration through the cornea and augments the therapeutic efficiency of entrapped drugs. Also, the optimum particle size allows the formation of a kinetically stable system that prevents particle sedimentation and/or aggregation (Vo et al., 2020). As depicted in Table 2, all prepared BS- loaded cubosomal dispersions showed a small PS ranging from 121.33 ± 19.98 to 260.37 ± 15.35 nm.

ANOVA analysis of the PS data indicated that model terms (A, C, AC, BC) had a significant effect (*P* < 0.05), while terms (B and AB) had no significant effect (*p* = 0.373 and 0.516, respectively) on PS. The polynomial equation connecting different factors and interactions in terms of coded values for PS was:

(5) Mean PS = +175.24 −14.94 A +2.45 B +20.67 C +1.78 AB −24.85 AC +21.7 BC

Accordingly, from the regression coefficients, PVA concentration (C) influenced positively while GMO concentration (A) affected negatively on the PS.

Results showed that an increase in the PVA concentration could cause clumping of the nanoparticles, initiating the growth in the particle size (Soleimani et al., 2016). High concentrations of PVA led to the higher viscosity of the external aqueous phase, triggering the particle size to increase due to decline in the net shear stress (Nawaz et al., 2021). However; the increase in the ratio of GMO: poloxamer 407 may cause a decrease in the accumulation of the cubosomes and the formed cubosomes size decreases (Eldeeb et al., 2019).

### 3.5. Effect of formulation variables on the mean PDI

PDI values were employed as an indication of the uniformity and overall size distribution of the prepared dispersions. A low PDI value approaching zero achieves a homogeneous dispersion with a narrow PS range. Conversely, a value that is much closer to 1 indicates a highly polydisperse dispersion (Naguib et al., 2021). In this study, the PDI values were ranging from 0.19 ± 0.02 to 0.48 ± 0.03 (Table 2) which represents the polydispersity of the prepared nanosystems.

The polynomial equation relating the influence of the formulation parameters for PDI in terms of coded values was:

(6) PDI = 0.343 −0.012 A +0.02 B +0.048 C −0.06 AB +0.044 AC −6.042 BC

As illustrated per equation, input factors such as concentrations of P407 (A) and PVA (C) exhibited a positive effect resulting in heterogeneous dispersions. In contrast, GMO concentration (B) had a negative effect and increasing this input parameter led to a narrow homogeneous distribution of the particle size.

The increase in the PDI values, upon increasing levels of Pluronic, might be associated with the formation of a vesicular micrometer structure which consequently led to a higher PDI value in case of high concentration of P407 (Tomoda et al., 2015). Parallel outcomes were stated, where diverse ultrastructure with altering P407 concentrations in monoolein dispersions were noted and examined (Wörle et al., 2007; Hakeem et al., 2020). It is reported that high Pluronic concentration produced diverse morphologies in the formed dispersion with self-aggregation and multiple forms initiation, causing the dispersion more heterogeneous and high value of PDI (Li et al., 2019; Al-Mahallawi et al., 2021). Furthermore, more heterogeneous dispersions are formed with an increase in PVA amounts. This could be explained that the high concentration of PVA attributed to an increase in PS which in turn is reflected in the PDI of the dispersion (Hakeem et al., 2020).

Higher amounts of GMO decreased PDI values, as the lipophilic GMO was able to surround the polymeric particles; forming a lipid coat that prevented the aggregation of the particles, thus diminishing PDI (Abdou et al., 2019). Similar findings were reported where the higher amounts of GMO were linked to obtaining smaller PS with smaller PDI (Kamel et al., 2019; Abd-Elsalam and Ibrahim 2021).

### 3.6. Selection and validation of BS-loaded cubosomal dispersions

Optimization was based on the highest values for ZP (as an absolute value)) and EE% as well as the lowest values of PS and PDI. The best achieved formulation from the experimental design with the desirability factor 0.822 was C1 that met the predetermined restraints. This optimum formula was prepared and processed for additional examinations. Moreover, Table 2 demonstrates the values of PS, ZP and PDI for the optimum formula (C1) with lacking hyaluronan. No significant difference was revealed except for the values of EE% and ZP (α < .05). The significant increase in the EE% may be attributed to the action of HA on increasing the viscosity of the inner core of the nanoparticles and the aqueous environment surrounding the lipid bilayers along with a subsequent reduction in the drug leakage from the nanoparticles (Kutbi et al., 2021). Considering the highly negative ZP, this might be justified due to the incorporation of negatively charged HA, causing extensive adsorption on the cubosomal surface. Previous findings stated that the increase in HA concentration first neutralized, then reversed the zeta potential of positively charged nanoparticles (Wadhwa et al., 2010; Tran et al., 2014).

### 3.7. Further in vitro characterization of optimal bromfenac cubosomal (OBC)

#### 3.7.1. Short term physical stability study

Samples from stored dispersions showed no recognized aggregation or change in their physical appearance after 45 and 90 days in both 4 °C and 25 °C. The values of EE%, ZP, PS and PDI for fresh and stored OBC are shown in Table 3. No significant change (*p* > 0.05 for all values) was observed in all studied responses. Therefore, it can be concluded that the storage at the specified conditions exhibited acceptable stability and also confirmed suitable homogeneity with high potential for corneal permeation (Sayed et al., 2021).

**Table 3.** Effect of storage conditions on the physical properties of optimum BS-loaded cubosomes (OBC) after time intervals (45 and 90 days) at both 4 and 25 ± 3 °C.

Parameter	fresh OBC	OBC after 45 days at 4 °C	OBC after 45 days at 25 °C	OBC after 90 days at 4 °C	OBC after 90 days at 25 °C
EE%	61.66 ± 1.01	62.4 23 ± 3.59	61.88 ± 3.54	60.56 ± 4.34	60.45 ± 4.58
ZP (mV)	30.80 ± 0.61	30.71 ± 3.31	30.11 ± 4.52	29.96 ± 3.38	29.88 ± 4.51
PS (nm)	149.3 ± 15.24	152.64 ± 9.81	150.65 ± 6.39	153.13 ± 7.46	154.71 ± 9.17
PDI	0.21 ± 0.02	0.23 ± 0.05	0.23 ± 0.03	0.22 ± 0.07	0.23 ± 0.09

Data presented as mean ± SD (*n* = 3).

**Abbreviations:** OBC, optimum bromfenac-loaded cubosomes; EE%, entrapment efficiency percent; ZP, zeta potential; PS, particle size; PDI, polydispersity index.

#### 3.7.2. Refractive index and pH measurement

The light refractive index of the OBC formulation was found to be 1.336 which is within the acceptable range (less than 1.5), causing no discomfort or blurred vision. Also, the pH value of OBC was determined (7.18 ± 0.11), indicating the suitability for its ocular use (pH of tears = 7.4) (Fouda Nagwa Hussein et al., 2018).

#### 3.7.3. Morphology of OBC

**Figure 2.**
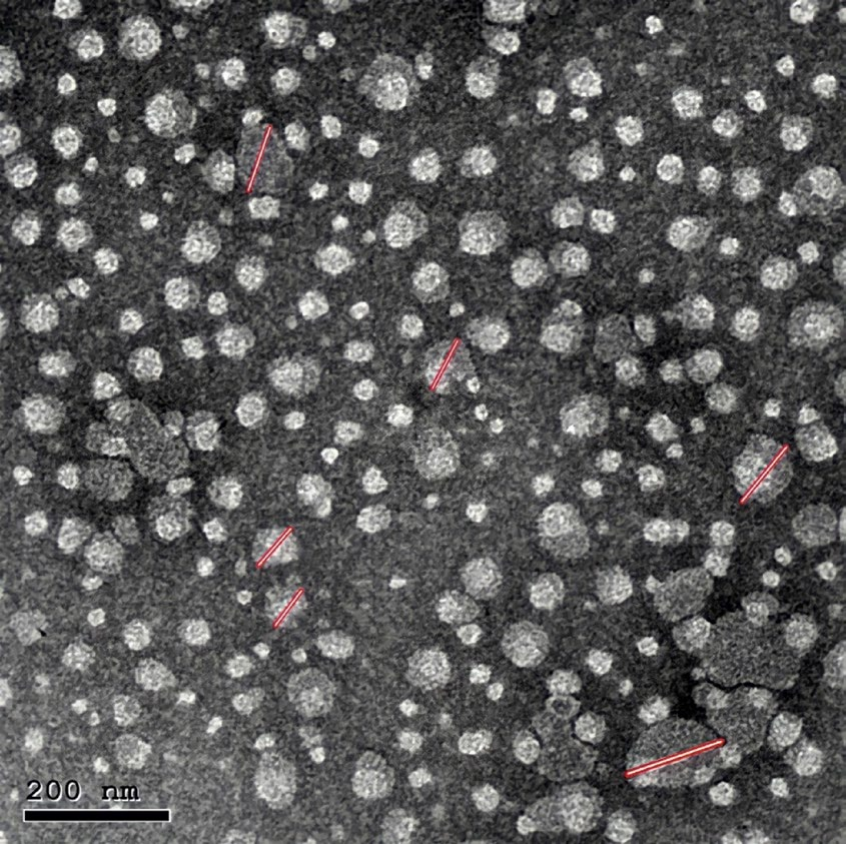
Surface morphology of optimized bromfenac-loaded cubosomes (OBC).

A representative TEM photograph of the selected OBC dispersion was depicted in Figure 2. The electron microscope image shows the formation of cubic phase nanocrystals with the typical characteristic structure of cubosomes. It was also observed that the formation of spherical shape particles without aggregation with uniform distribution assuring its stability. The majority of cubosomal particles determined by TEM was in the nano range and had a smaller particle diameter compared to the average particle size (Z-average) values measured by Zetasizer (Table 2). This may be attributed to the process of sample drying just before TEM imaging.

### 3.8. Ex vivo and in vivo characterization of OBC

#### 3.8.1. Transcorneal drug permeation studies

**Figure 3.**
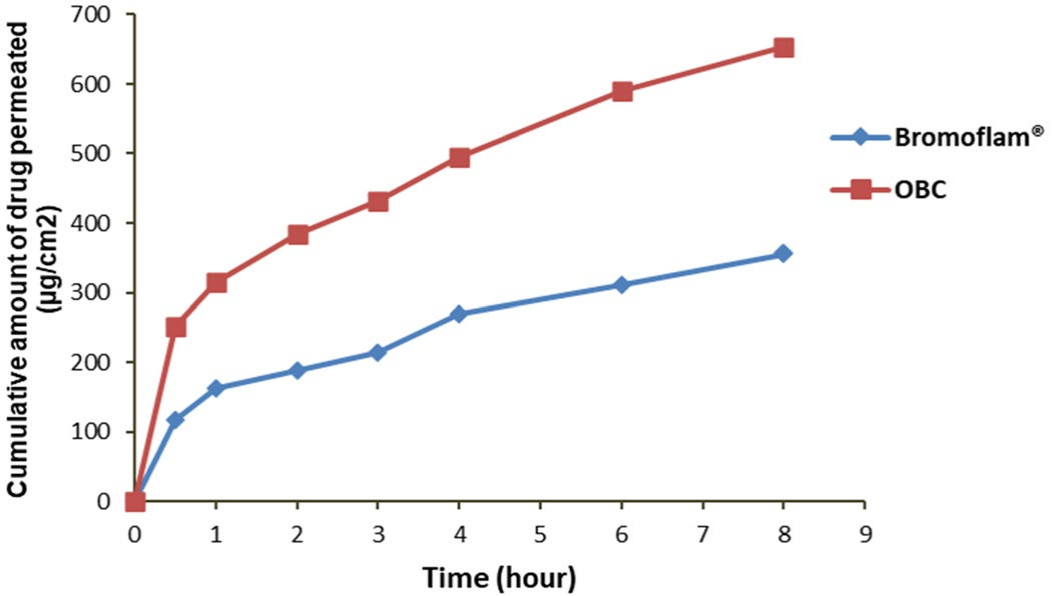
Cumulative amounts of drug permeated through the goat cornea from Bromoflam^®^ eye drops and optimized bromfenac-loaded cubosomes (OBC). Data expressed as mean ± SD (*n* = 3).

Comparative ex vivo permeation was conducted to investigate the permeability potential of BS from the control Bromoflam^®^ eye drops and optimized cubosomal dispersion through excised goat cornea for a period of 8 h. Biological tissues may have the ability to improve drug permeation while also facilitating drug absorption. Furthermore, the main component of cubosomal dispersion, GMO, has an excellent permeability enhancer action via corneal tissue. The findings of this study are consistent with numerous previous studies that have shown that cubosomes can enhance corneal permeability and drug bioavailability, allowing them to successfully treat ocular diseases (Younes et al., 2018; Gaballa, El Garhy, Abdelkader 2020; Nasr Mohamed et al., 2020). The complete permeation profile for both formulations was described in Table 4 and Figure 3 indicating the cumulative percentage of BS permeated through the cornea at each time point. The results showed that the quantity of Bromfenac permeated through the cornea and deposited in the corneal tissues was significantly (*p* < 0.05) greater than that from the market product. When compared to the marketed eye drops, the permeability coefficient (Kp) and the corneal deposition were increased significantly (1.8-fold and 10.66-fold, respectively). In addition, the enhancement ratio of OBC formula was increased 1.89-folds. Consequently, OBC formula was suggested to be the most promising system in improving the ocular efficiency parameters of BS. The superior permeability profile of OBC dispersion through the goat cornea may be attributed to the depot formation in close proximity of cornea which released the drug in a sustained manner, in addition to improved endocytosis of cubosomes by corneal epithelial tissue (Gade et al., 2019).

**Table 4.** Permeability parameters of BS after application of Bromoflam® eye drops and OBC dispersion through the goat cornea.

Permeability parameters	Bromoflam®	OBC
Total amount of bromfenac permeated per unit area in 8 h (µg/cm2)	451.72 ± 1.42	833.50 ± 2.11
Apparent permeability coefficient (Kp)(cm/h)	0.06 ± 0.043	0.11 ± 0.06
Corneal deposition (µg/cm2)	22.17 ± 1.72	235.84 ± 4.25
Enhancement ratio	–	1.89

Data expressed as mean ± SD (*n* = 3).

#### 3.8.2. Confocal laser scanning microscopy (CLSM)

**Figure 4.**
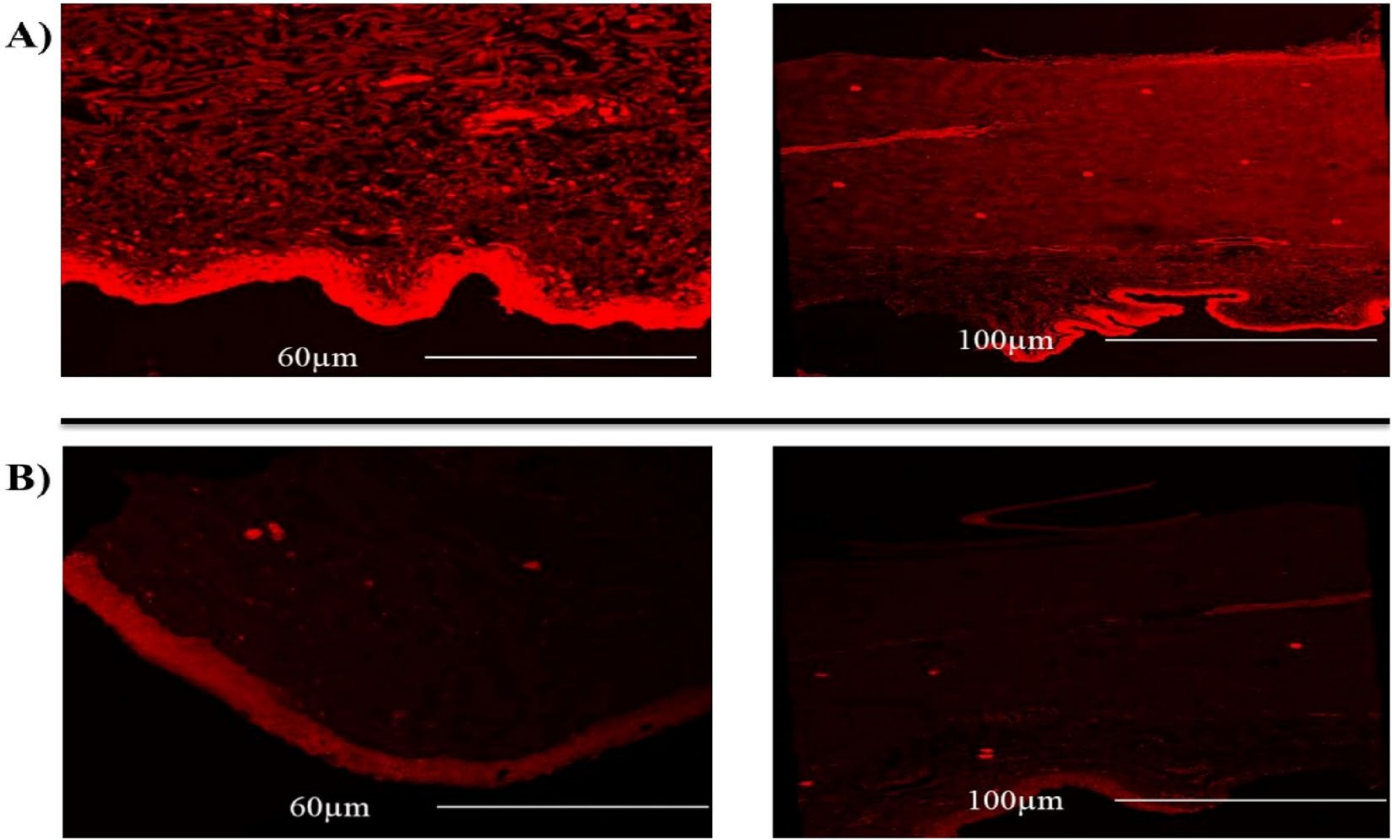
Confocal laser scanning micrographs of existed goat cornea at different penetration depth (60 -100 µm) following treatment with A) cubosomal disperion B) for Rhodamine B dye solution.

CLSM was assessed to estimate the penetration ability of the cubosomal dispersion compared to the standard rhodamine solution. The CLSM images of longitudinal corneal specimen for the dye solution and the best achieved formulae were showed in Figure 4. The scanned images showed the highly penetration and deposition of fluorescence along the deeper layers of the corneal tissue of C1 compared to that of the dye solution into great extent up to 100 µm depth in the cornea with a high intensity of the dye that supported the ex vivo permeation results. The smaller size of nanoparticles could be the major reason for the previous illustrations that might assist the deep penetration in the corneal tissue as previously discussed.

#### 3.8.3. In vivo assessment of ocular bioavailability

**Figure 5.**
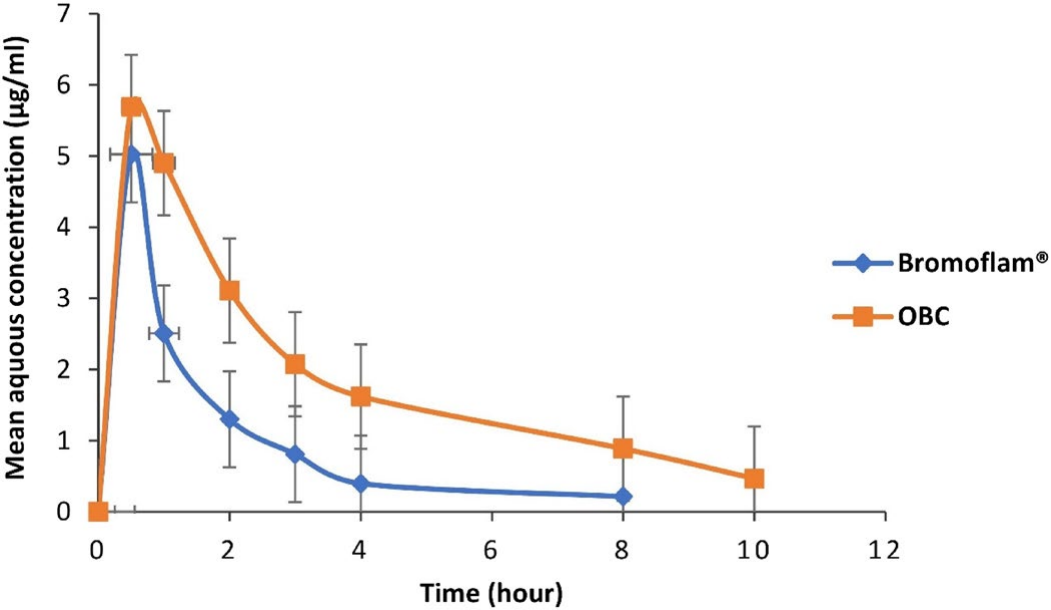
Mean aqueous humor concentration versus time profiles of bromfenac after topical single administration of Bromoflam® eye drops and optimized bromfenac-loaded cubosomes (OBC) at a dose of 0.025 mg/kg in New Zealand White male rabbits.

The mean aqueous humor versus time profiles of BS obtained following topical ophthalmic administration of the commercial eye drops and OBC dispersion (dose of 0.025 mg/kg) are given in Figure 5.

The non-compartmental analysis was performed to determine various pharmacokinetic parameters. Concentration at peak (C_max_), time to reach peak concentration (T_max_) half-life (T_1/2_), and mean residence time (MRT) were reported directly from the observed data. The area under the aqueous humor concentration-time curve (AUC) versus time was determined by the linear trapezoidal with linear interpolation method. The total area from time zero to the last measurable concentration (AUC_0-tlast_) and the total area from time zero to infinity (AUC_0-∞_) were estimated. Individual aqueous humor parameters for each eye were analyzed. All pharmacokinetic parameters were reported as mean ± SD and were represented in Table 5 and Figure 5.

**Table 5.** Ocular pharmacokinetic parameters in aqueous humor obtained after topical single administration of Bromoflam® eye drops and OBC dispersion at a dose of 0.025 mg/kg in New Zealand White male rabbits.

Parameters	Parameter values (Mean ± SD)	
	Bromoflam®	OBC
**C_max_ (μg/mL)**	5.02 ± 0.41	5.69 ± 0.32
**T_max_ (h)**	0.50 ± 0.00	0.50 ± 0.00
**AUC _0-t last_ (μg.h/mL)**	7.93 ± 2.28	18.88 ± 6.13
**AUC _0-∞_ (μg.h/mL)**	8.69 ± 3.15	21.23 ± 5.36
**T_1/2_ (h)**	2.46 ± 0.11	3.48 ± 0.15
**MRT (h)**	1.97 ± 0.46	3.16 ± 0.68

Data are presented as mean ± SD (*n* = 5).

**Abbreviations:** OBC, optimum bromfenac-loaded cubosomes; C_max_, peak plasma concentration; T_max_, time to reach maximum peak; AUC, area under the curve; h, hours; T_1/2_, half-life; MRT, mean residence time; SD, standard deviation.

The OBC dispersion had a peak concentration of 5.024 μg/mL, which was lower that for the marketed eye drops (5.69 μg/mL). Both treatments reach a peak concentration after 30 min. BS embedded in OBC dispersion had a half-life of 3.482 h, which was 1.42 times longer than that of the marketed eye drops group (2.458 h). The OBC dispersion exhibited a more than 150% improvement (3.162 h) in the mean retention time as compared to that of the market product (1.966 h). This is most likely due to the lipid components, GMO and P407 encapsulating BS, which prevented the burst release of BS and ensured its sustained release in the cornea. Furthermore, the viscosity and adhesive properties of GMO enable the cubosomes to attach to the ciliary muscle and conjunctival sac, enhancing the residence time of BS and assuring the drug release at the site of administration consistently (Huang et al., 2017). When the proportion of BS absorbed increased, the extent of BS that drains across the nasolacrimal duct into the systemic blood circulation was reduced. As a result, the systemic side effect of BS may be diminished.

The concentration of BS from the marketed eye drops was detectable up to 8 hours, with the area under the curve (AUC) of 7.929 μg.h/mL. Alternatively, the concentration of BS from OBC dispersion was detectable up to 10 hours, with an AUC of 18.88 μg.h/mL. Also, the AUC_0-∞_ value of OBC dispersion (21.226 μg.h/mL) was 2.44-fold increase compared to that of the market product (8.694 μg.h/mL), *P* < 0.05. The ratio of AUC_0-tlast_ to AUC_0-∞_ for the marketed eye drops and OBC dispersion was found to be higher than 0.91 and 0.89, respectively, suggesting the utilized approach was sensitive to the sample analysis until around 90% of BS are discharged from the aqueous-humor.

It can be concluded from the present findings that the intraocular permeation and the retention time of BS in the anterior ocular tissues and aqueous humor were significantly improved with a higher drug bioavailability for OBC treatment.

#### 3.8.4. Histopathological examination for ocular irritation

**Figure 6.**
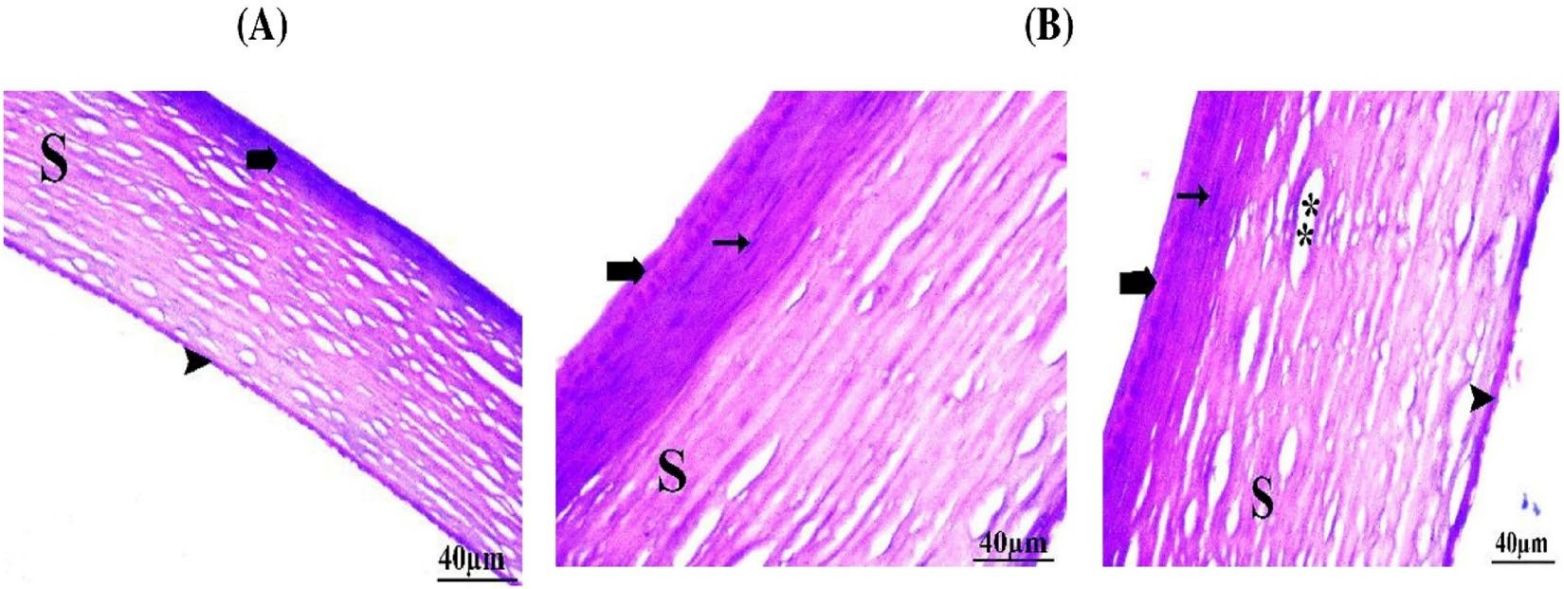
Photomicrographs showing histopathological sections of excised rabbit cornea treated with A) normal saline (control) and B) optimized bromfenac-loaded cubosomes (OBC) comparing epithelium (bold arrow), inner endothelium (arrowhead), and stroma (S), wide space (double asterisk), keratocyte (arrow).

Histopathological test is essential to examine the ocular tolerability and safety of the topically applied OBC dispersion versus normal saline (negative control). Histological examination was performed for the corneal tissues of rabbit after successive of administration of OBC formula. Regarding the treated cornea group, there were mild unsignificant histopathological alterations (Figure 6B) represented in thin epithelium, intact inner endothelium, and more or less organized stroma, and regular keratocytes in comparison to the negative control group (Figure 6A). Lack of histological insufficiency or irritation caused by OBC formulation proposed the good biocompatibility of the applied excipients used and the safe use of cusbosomes as carrier for the ocular delivery of drugs.

## 4. Conclusion

Hyaluronan enriched cubosomal dispersions loaded with an anti-inflammatory drug, BS, were investigated as an ocular drug delivery system. Incorporating hyaluronan and PVA into developed formulations was superior in terms of improving the physical characterization and stability of BS-loaded cubosomes as well as their palliative impact on alleviating dry eye syndrome and ocular inflammation. Eight BS-loaded cubosomes were successfully prepared and statistically optimized utilizing 2^3^ full factorial design. The optimized formulation had high EE% and negative ZP, in addition to exhibiting spherical nanosized particles and excellent dispersibility. It exhibited considerable stability for 90 days upon refrigeration at 4–8 °C. It demonstrated no ocular irritation or blurring in vision as reflected by pH and refractive index measurements. Moreover, a significantly higher ex vivo transcorneal permeation and better distribution to the cornea were achieved by the optimized formula, when compared to the marketed eye drops (control). Confocal laser microscopy images also ensured the deeper penetration of cubosomal dispersions through goat cornea. Subsequently, improved ocular bioavailability of the optimized formula was confirmed by the increased AUC_0-t_, AUC_0-∞_ and mean residence time, when compared with the marketed eye drops. Furthermore, the histopathological study confirmed the safety of the optimized formula, exerting no deleterious influence on rabbit corneal structure and integrity. In conclusion, developed cubosomes have a greater potential as a safe and effective delivery of BS for treating ocular diseases.
